# Hemorrhagic Stroke in Atrial Fibrillation: Trends in Incidence, Case Fatality, and Prior Oral Anticoagulation

**DOI:** 10.1161/JAHA.124.040360

**Published:** 2025-06-11

**Authors:** Paula Tiili, Mika Lehto, Olli Halminen, Jari Haukka, Ossi Lehtonen, Miika Linna, Jarno Satopää, Miikka Korja, Mika Niemelä, Janne Kinnunen, Aapo L. Aro, Pirjo Mustonen, Juha Hartikainen, K. E. Juhani Airaksinen, Jukka Putaala, the FinACAF study group

**Affiliations:** ^1^ Primary Health Care Unit Helsinki University Hospital Helsinki Finland; ^2^ University of Helsinki Helsinki Finland; ^3^ Jorvi Hospital, Department of Internal Medicine Helsinki University Hospital Espoo Finland; ^4^ Department of Industrial Engineering and Management Aalto University Espoo Finland; ^5^ University of Eastern Finland Kuopio Finland; ^6^ Department of Neurosurgery Helsinki University Hospital Helsinki Finland; ^7^ Department of Neurology Helsinki University Hospital Helsinki Finland; ^8^ Heart and Lung Center Helsinki University Hospital Helsinki Finland; ^9^ Turku University Hospital Turku Finland; ^10^ University of Turku Turku Finland; ^11^ Kuopio University Hospital Kuopio Finland

**Keywords:** anticoagulation, atrial fibrillation, epidemiology, hemorrhagic stroke, Intracranial Hemorrhage, Atrial Fibrillation

## Abstract

**Background:**

Despite increased atrial fibrillation (AF) and oral anticoagulation (OAC) use among patients with intracerebral hemorrhage, trends in hemorrhagic stroke (HS) incidence in AF remain unclear. We examined recent epidemiological trends, considering advances in stroke prevention and an aging population.

**Methods and Results:**

A Finnish nationwide study using linked administrative registries followed 168 121 patients with incident AF (2009–2017) until the first‐ever intracerebral or subarachnoid hemorrhage, death, or study end (November 2018). HS incidence rates, the proportion of fatal cases (case fatality rate %), and anticoagulant purchases within 90 days before HS were analyzed. Temporal trends were assessed using logistic and Poisson regression. A total of 1890 patients with AF (53% women, median age 80.2) experienced their first‐ever HS. The average annual incidence rate was 2.82/1000 PY (95% CI, 2.72–2.92), remaining stable over 10 years (range 2.62–3.22). Age‐standardized incidence rates were 2.65 (2.48–2.82) for women and 2.94 (2.70–3.15) for men. The annual case fatality rate averaged 46.5% (43.9%–49.1%) without notable temporal or sex differences. Older age was associated with higher incidence rates and case fatality rate, with an increasing proportion of patients over 84. Prior OAC use increased: in 2009, 67% were not on OACs but by 2018, one third used warfarin, one third direct OACs, and only one third were without any OAC. No association was found between anticoagulation and HS case fatality.

**Conclusions:**

Among the population with incident AF, we observed a stable first‐ever HS incidence rate and short‐term case fatality over the past decade despite a marked increase in prior oral anticoagulation use and aging.

**Nonstandard Abbreviations and Acronyms**
CFR: case fatality rate
DOAC: direct oral anticoagulant
FinACAF: Finnish AntiCoagulation in Atrial Fibrillation study
HS: hemorrhagic stroke
ICH: intracerebral hemorrhage
IR: incidence rate
OAC: oral anticoagulants, oral anticoagulation regimen

**CLINICAL PERSPECTIVE**

**What Is New?**

- There is paucity on research how the population aging and recent changes in stroke prevention manifest in hemorrhagic stroke occurrence and severity in the population with atrial fibrillation (AF).
- Over the past decade, including stronger recommendations for oral anticoagulant (OAC) usage in guidelines and the introduction of direct oral anticoagulants, we observed a stable incidence rate of hemorrhagic stroke and subsequent 30‐day case fatality rate in a nationwide unselected population with AF, despite an increased proportion of patients with OAC purchases within the previous 90 days.
- We did not observe associations between hemorrhagic stroke case fatality and prior OAC purchases, whereas higher age was associated with both higher incidence and case fatality, and the proportion of patients >84 years of age increased.

**What Are the Clinical Implications?**

- An aging population with AF and the increased use of OACs reported by other studies have not led to an increase in the incidence rate of hemorrhagic stroke or subsequent case fatality in this real‐life, unselected population with AF.
- Along with an assumed increase in AF prevalence, our findings suggest a growing absolute number of older patients with AF will suffer OAC‐related hemorrhagic stroke, as well as an increasing number of survivors of hemorrhagic stroke with AF in the future.

Hemorrhagic stroke (HS), including both intracerebral hemorrhage (ICH) and subarachnoid hemorrhage (SAH), is known for its high morbidity and mortality and contributes 15% to –40% of all stroke incidences.^1^ Patients with atrial fibrillation (AF) constitute a specific population with increased risk of HS, as they are typically older and have an indication for long‐term oral anticoagulation (OAC).^2^ Additionally, these patients commonly have multiple comorbidities associated with elevated HS risk, including amyloid angiopathy and small‐vessel disease of the brain, often coupled with frailty, polypharmacy, and functional impairments.^3^, ^4^, ^5^, ^6^

As higher age is a major predictor of AF, the prevalence of AF continues to rise with an aging population.^7^, ^8^ The overall prevalence of AF in adults has been reported to be as high as 5.2% and 23% among those >75 years of age and is anticipated to further increase.^8^ Simultaneously, during the past decade, direct oral anticoagulants (DOACs) have been introduced as an option for thrombosis prophylaxis. With a lower risk of intracranial bleeding, along with simpler administration and similar effectiveness compared with warfarin, their use has drastically increased within the population with AF.^9^, ^10^, ^11^

Recent studies in the general population have shown relatively stable or decreasing HS incidence^1^, ^12^, ^13^, ^14^ and mortality rate^12^, ^15^, ^16^, ^17^ but an increased proportion of individuals with AF^12^, ^18^ and those using some OAC.^12^, ^18^, ^19^ However, whether the incidence and severity of HS have recently changed in the population with AF remains unclear, especially considering aging and recent changes in thrombosis prophylaxis. Population level studies in AF on HS epidemiology are sparse, and although some data, mainly from the pre‐DOAC era, exist,^20^, ^21^, ^22^, ^23^, ^24^, ^25^, ^26^, ^27^ recent studies primarily involved only individuals using some form of OAC, excluding those without OAC in use.^28^, ^29^, ^30^, ^31^, ^32^ Thus, we aimed to use a nationwide, unselected cohort with AF^8^ to investigate temporal trends in HS incidence, 30‐day case fatality, and OAC use before HS (as a major exposure to HS) from 2009 to 2018, encompassing the introduction of DOACs.

## METHODS

### Study Design and Population

This is a population‐based longitudinal study using Finnish nationwide administrative health registries. The FinACAF (Finnish AntiCoagulation in Atrial Fibrillation) study linked all nationwide registries to establish a comprehensive database of patients with AF in Finland 2004 to 2018. The construction of the FinACAF database is thoroughly explained elsewhere.^33^ For this substudy, to allow a minimum of a 5‐year look‐back period for exclusion,^34^, ^35^, ^36^, ^37^ we included patients if they had incident AF or atrial flutter (*International Classification of Diseases, Tenth Revision* [*ICD‐10*], code I48)^8^ recorded between January 2009 and November 2017, without any diagnosis of intracranial hemorrhage in hospital or primary care registries before cohort entry date. We further excluded patients who experienced incident AF and HS on the same day. The cohort selection flow chart is provided in Supplemental Figure 1. Given the sensitive nature of the data used, researchers can submit data access requests to the Finnish national register holders through Findata (https://findata.fi/en/).

The observed outcome was HS, including nontraumatic SAH and ICH (*ICD‐10* codes I60, I61)^1^ in the National Hospital Care Registry or as the main cause of death in the National Death Registry. This is in line with the previously published definition of HS as a nontraumatic bleeding from ruptured cerebral vessel to brain parenchyma or subarachnoid space causing stroke symptoms or brain lesion.^1^, ^38^

To minimize the inherent risk of misclassification when using administrative data, and also to include nonaneurysmal SAHs, we chose to use *ICD‐10* codes without detailed classification by location or source of bleeding, including all *ICD‐10* I60* and I61* codes in our case identification. Patients identified through the hospital care registry had to have over a 2‐day hospitalization with HS as the primary diagnosis, whereas those recognized based on the cause of death could have a shorter hospitalization before death. To distinguish actual HS from ischemic stroke with hemorrhage and from traumatic intracranial bleedings, we censored patients at the time of HS diagnosis if it was associated with such event diagnoses. The flow chart for case identification and the registries used are presented in Figure S2. The Finnish Care Registry and National Causes of Death registry data have been validated previously.^39^, ^40^

The follow‐up ended upon the occurrence of the earliest of the following events: the outcome event, exclusion due to a diagnosis of HS associated with traumatic intracranial bleeding or ischemic stroke, death from causes other than the outcome event, or reaching the end of the observation period on November 30, 2018. Cases were followed for an additional 30 days from the event date to assess proportion of fatal cases within the time frame.

For all cases, we documented any OAC purchases within 90 days before the HS event, and patients with such purchases were considered OAC users by the time of the event. Additionally, we assessed CHA_2_DS_2_‐VASc and modified HAS‐BLED scores at the cohort entry and for cases by the time of the event. Supplemental Table 1 shows *ICD‐10* codes used in the study and Supplemental Table 2 shows Anatomical Therapeutic Chemical Classification used for medication identification.

The study is reported according to the Strengthening the Reporting of Observational Studies in Epidemiology guidelines and checklist with Reporting of Studies Conducted Using Observational Routinely Collected Data statement can be found from the data supplement. The study follows the principles of the Declaration of Helsinki (revised in 2013) and has received approval from the Ethics Committee of the Medical Faculty of Helsinki University and permissions from pertinent Finnish register holders. According to Finnish legislation, informed consent was unnecessary for this retrospective registry‐based study.

### Statistical Analysis

We reported frequencies for variables as absolute numbers and, if categorical, as percentages and as medians with interquartile ranges for continuous variables. We calculated first‐ever HS incidence rates (IRs) for the entire cohort and stratified by sex, age groups (20–64, 65–74, 75–84, and ≥85 years) and calendar years. For temporal analyses, we also categorized cohort entry years and event years (2009–2011, 2012–2014, and 2015–2017/2018) into 3 categories reflecting the introduction of DOACs in the country. The IRs were calculated as the rate of events over time at risk and reported per 1000 patient‐years. In addition, we calculated age‐standardized IRs via direct standardization, with the cohort baseline population used as the reference population. The 30‐day case fatality rate (CFR) was calculated as the proportion of fatal cases of all cases within a 30‐day window and reported as percentages.

Pearson's chi‐square, Fisher's, Mann–Whitney *U*, and Kruskal–Wallis tests were used to compare differences in categorical and continuous variables. Poisson regression was used to evaluate time trends in the incidence rates, and logistic regression was used to estimate trends and associations with the CFR. We calculated 95% CIs for the estimations and considered *P* values <0.05 statistically significant. The main analyses were also conducted separately for ICH and SAH and are reported in data supplement. Statistical analyses were performed with IBM SPSS (version 28.0.0.0; IBM Corporation, Armonk, NY) and Rstudio (R Core Team, 2023).

## RESULTS

### Population and Patient Characteristics

In total, 168 121 patients (50% women, median age 74.3 years) were included in the initial cohort of patients with incident AF or atrial flutter, contributing to 676 681 patient‐years. Women entered the cohort at an older median age compared with men, 78.3 (interquartile range, 69.6–85.1) versus 70.0 (61.2–78.7) years (*P*=0.001). Among women, 39% were <75 years and 25% were >84 years, whereas among men, 65% were younger than 75 years and 10% were >84 years. Baseline characteristics over the cohort entry years are presented in Table S3.

During the observation period, altogether 1890 patients (53% women, median age 80.2 years) suffered their first‐ever HS (SAH, n=252; ICH, n=1638). Patient characteristics for individuals experiencing their first‐ever HS are shown in Table 1, and the breakdown by event year is shown in Table S4. Among those with HS, the proportion of sexes remained similar, but women were on average 7 years older than men. Over the years, the overall proportion of patients >84 years of age increased from 17% in 2009 to 36% in 2018 (Figure S3). Most patients had a CHA_2_DS_2_‐VASc score ≥2. For 42% of the patients, the HAS‐BLED score was ≥3 (Table 1).

**Table 1:** Characteristics of Patients With Atrial Fibrillation and First‐Ever Hemorrhagic Stroke Identified Between 2009 and 2018, Overall and Compared Between Sexes

Characteristic	Overall N=1890	Female N=1003	Male N=887	*P* value
Age, y	80.2 (72.7–86.1)	83.0 (77.3–87.8)	75.7 (69.5–82.9)	<0.001
Age group, y				<0.001
20–64	155 (8.2%)	37 (3.7%)	118 (13%)	
65–74	458 (24%)	158 (16%)	300 (34%)	
75–84	730 (39%)	417 (42%)	313 (35%)	
≥85	547 (29%)	391 (39%)	156 (18%)	
CHA_2_DS_2_‐VASc	4.0 (3.0–6.0)	5.0 (4.0–6.0)	4.0 (3.0–5.0)	<0.001
CHA_2_DS_2_‐VASc categories				<0.001
0	16 (0.8%)	0 (0%)	16 (1.8%)	
1	61 (3.2%)	1 (<0.1%)	60 (6.8%)	
≥2	1813 (96%)	1002 (100%)	811 (91%)	
HAS‐BLED	2.0 (2.0–3.0)	2.0 (2.0–3.0)	2.0 (2.0–3.0)	0.499
HAS‐BLED categories				0.109
<3	1103 (58%)	603 (60%)	500 (56%)	
≥3	787 (42%)	400 (40%)	387 (44%)	
Prior OAC purchase				0.219
Direct OAC	218 (12%)	117 (12%)	101 (11%)	
Warfarin	821 (43%)	453 (45%)	368 (41%)	
None	851 (45%)	433 (43%)	418 (47%)	
Cohort entry year				0.447
2009–2011	781 (41%)	404 (40%)	377 (43%)	
2012–2014	697 (37%)	370 (37%)	327 (37%)	
2015–2017	412 (22%)	229 (23%)	183 (21%)	
Event year				0.085
2009–2011	179 (9.5%)	81 (8.1%)	98 (11%)	
2012–2014	528 (28%)	287 (29%)	241 (27%)	
2015–2018	1183 (63%)	635 (63%)	548 (62%)	

Numbers presented are median (interquartile range) or number of cases (%).OAC indicates oral anticoagulant.

### Anticoagulant Use Preceding HS

Among patients who suffered HS, the percentage of those without any OAC prior in use decreased from 61% in 2012 to 33% in 2018. Warfarin usage peaked at 53% in 2014 and then decreased to 33% in 2018. The proportion of patients with DOACs began to rise in 2015, reaching 33% in 2018. Among all patients who experienced an HS event, 55% were on OACs, 43% were on warfarin and 12% were on DOACs, without a significant difference in OAC use between the sexes (Table 1). However, the uptake of DOACs was more prominent among men compared with women (Figure S4).

### Incidence Rates of HS

**Figure 1:**
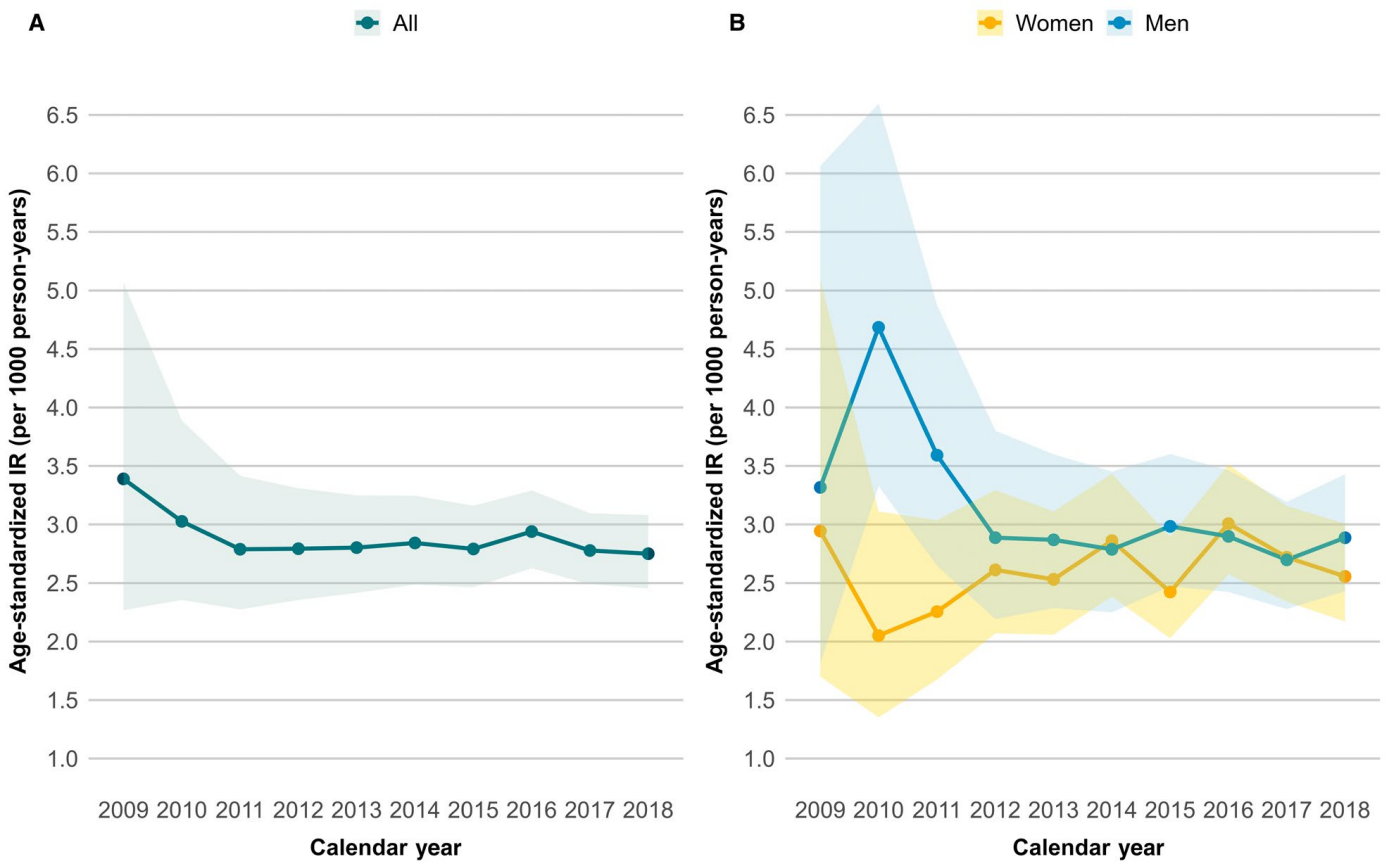
Temporal trend of the age‐standardized first‐ever hemorrhagic stroke incidence rate among patients with atrial fibrillation. **A**, All patients; **B**, Stratified by sex. The shaded areas denote 95% CIs. IR indicates incidence rate.

With an average annual crude IR of 2.82 (95% CI, 2.72–2.92) per 1000 patient‐years and an annual IR range of 2.63 to 3.22, we found no obvious trends in the incidence rate of HS over time. Age was strongly associated with the IR of HS, with patients aged 85 years and older having a 5‐fold higher rate than those <65 years. The crude IR was significantly higher for women than for men, but after age standardization IR was lower for women than for men, 2.65 (2.48–2.82) versus 2.94 (2.7–3.15). The temporal trend of age‐standardized HS IR by sex is shown in Figure 1. Table 2 presents IRs by patient characteristics and time periods, along with adjusted IR ratios. Additionally, Supplemental Table 5 shows the results stratified by sex and Figure S5 shows the annual crude incidence rates with corresponding prior OAC use (Table 2).

**Table 2:** Crude Incidence Rates Per 1000 Patient‐Years and Adjusted Incidence Rate Ratios Between 2009 and 2018 in the Overall Cohort. Separate Models to Estimate Temporal Trends by Calendar Years and Time Periods

Characteristic	Events (N)	PY	IR (95% CI)	IRR (95% CI)*	*P* value*
All patients	1890	676 681	2.79 (2.67–2.92)	…	…
Women	1003	331 869	3.02 (2.84–3.22)	Reference	
Men	887	344 812	2.57 (2.41–2.75)	1.12 (1.02–1.23)	0.020
Age group, y					
20–64	155	164 777	0.94 (0.80–1.10)	Reference	
65–74	458	188 567	2.43 (2.21–2.66)	2.61 (2.18–3.15)	<0.001
75–84	730	202 821	3.60 (3.34–3.87	3.93 (3.31–4.70)	<0.001
≥85	547	120 517	4.54 (4.17–4.94)	5.05 (4.22–6.08)	<0.001
Cohort entry years					
2009–2011	781	300 254	2.60 (2.41–2.79)	Reference	
2012–2014	697	247 869	2.81 (2.61–3.03)	1.10 (0.99–1.23)	0.088
2015–2017	412	128 556	3.20 (2.90–3.53)	1.27 (1.10–1.45)	<0.001
Calendar years				0.97 (0.95–1.0)†	0.018†
2009–2011	180	64 658	2.78 (2.39–3.22)	Reference	
2012–2014	528	193 289	2.73 (2.50–2.97)	0.92 (0.77–1.09)	0.330
2015–2018	1182	418 734	2.82 (2.66–2.99)	0.85 (0.71–1.01)	0.066

IR indicates incidence rate per 1000 patient‐years; IRR, incidence rate ratio; and PY, patient‐years.

* Model adjusted by sex, age group, and cohort entry and calendar year periods.

^†^ Model adjusted by sex, age group, and cohort entry and calendar years.

### Thirty‐Day Case Fatality of HS

**Figure 2:**
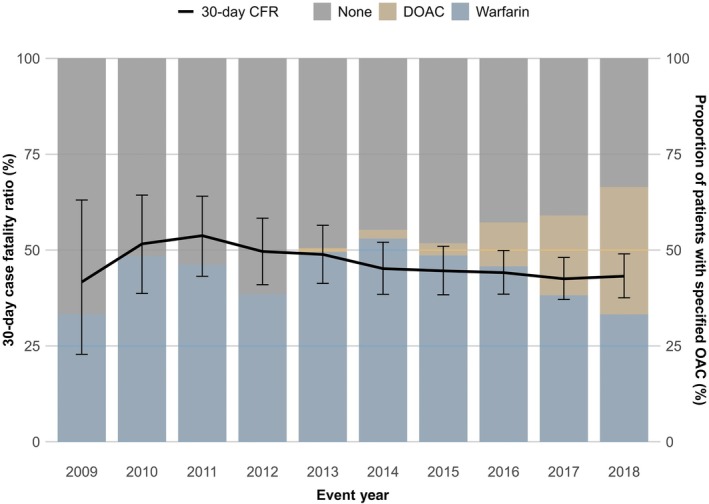
Temporal changes in the 30‐day case fatality rate after first‐ever hemorrhagic stroke among patients with atrial fibrillation and proportions of patients with oral anticoagulant purchase before the event. CFR indicates case fatality rate; DOAC, direct oral anticoagulant; and OAC, oral anticoagulant.

The annual average 30‐day case fatality rate peaked in 2011 at 53.8% and then gradually decreased to 43.2% in 2018. However, no significant temporal trend was observed (Figure 2). The average 30‐day CFR after HS was 45.4% (43.1%–47.7%), annually ranging from 41.7% to 53.8%. The 30‐day CFR was higher in women (48.5%, 45.3%–51.6%) than in men (42%, 38.7%–45.3%). After adjusting for patient characteristics, this sex difference was no longer significant. Higher age was significantly associated with higher mortality, ranging from 30.3% (23.3–38.3) in patients aged 20 to 64 years to 54.1% (49.8–58.3) in patients >84 years of age. We did not find significant associations between 30‐day CFR and prior OAC use in either univariate or multivariate analyses. Table 3 shows the 30‐day CFRs by prespecified time periods and patient characteristics, stratified by sex. Table 4 displays the adjusted odds ratios for assessing temporal trends in CFR after first‐ever HS among patients with AF. Additionally, Figure S6 illustrates annual CFRs by age groups with the 2 youngest groups combined due to low number of fatal cases in the youngest age group. By unadjusted logistic regression models, the trends presented were unsignificant.

**Table 3:** The 30‐Day Case Fatality Rate After First‐Ever Hemorrhagic Stroke Among Patients With Atrial Fibrillation by Case Characteristics (Subgroup) and Stratified by Sex

Subgroup	All		Women		Men	
	Death/cases	CFR% (95% CI)	Death/cases	CFR% (95% CI)	Death/cases	CFR% (95% CI)
All	858/1890	43.4 (43.1–47.7)	486/1003	48.5 (45.3–51.6)	372/887	41.9 (38.7–45.3)
Age group, y						
20–64	47/155	30.3 (23.3–38.3)	9/37	24.3 (12.4–41.6)	38/118	32.2 (24.1–41.5)
65–74	165/458	36.0 (31.7–40.6)	53/158	33.5 (26.4–41.5)	112/300	37.3 (31.9–43.1)
75–84	350/730	47.9 (44.3–51.6)	206/417	49.4 (44.5–54.3)	144/313	46.0 (40.4–51.7)
≥85	296/547	54.1 (49.8–58.3)	218/391	55.8 (50.7–60.7)	78/156	50.0 (42.2–57.8)
CHA_2_DS_2_‐VASc						
0	2/16	12.5 (2.2–39.6)	0/0	NA	2/16	12.5 (2.2–39.6)
1	25/61	41.0 (28.8–54.3)	0/1	NA	25/60	41.7 (29.3–55.1)
≥2	831/1813	45.8 (43.5–48.2)	486/1002	48.5 (45.5–51.6)	345/811	42.5 (39.1–46.0)
Modified HAS‐BLED						
0	3/19	15.8 (4.17–40.5)	0/3	NA	3/16	18.8 (5.0–46.3)
1–2	492/1084	45.4 (42.4–48.4)	289/600	48.2 (44.1–52.2)	203/484	41.9 (37.5–46.5)
≥3	363/787	46.1 (42.6–49.7)	197/400	49.2 (44.3–54.3)	166/387	42.9 (37.9–48.0)
Prior OAC purchase						
None	386/851	45.1 (42.0–48.8)	206/433	47.6 (42.8–52.4)	180/418	43.1 (38.3–48.0)
Warfarin	382/821	46.5 (43.1–50.0)	225/453	49.7 (45.0–54.4)	157/368	42.7 (37.6–47.9)
Direct OAC	90/218	41.3 (34.7–48.1)	55/117	47.0 (37.8–56.4)	35/101	34.7 (25.6–44.8)
Event year period						
2009–2011	93/180	51.7 (44.1–59.1)	40/81	49.4 (38.2–60.6)	53/99	53.5 (43.3–63.5)
2012–2014	251/528	47.5 (43.2–51.9)	149/288	51.7 (45.8–57.6)	102/240	42.5 (36.2–49.0)
2015–2018	514/1182	43.5 (40.6–46.4)	297/634	46.8 (42.9–50.8)	217/548	39.6 (35.5–43.8)

CFR indicates case fatality rate; and OAC, oral anticoagulant.

**Table 4:** Multivariable Logistic Regression Model to Access Temporal Trends in 30‐Day Case Fatality Rate After First‐Ever Hemorrhagic Stroke Among Patients With Atrial Fibrillation, for All and Stratified by Sex

Characteristics	All		Women		Men	
	OR (95% CI)	*P* value	OR (95% CI)	*P* value	OR (95% CI)	*P* value
Sex						
Female	Reference					…
Male	0.94 (0.76–1.17)	0.596				…
Age group, y						
20–64	Reference		Reference		Reference	
65–74	1.19 (0.79–1.80)	0.416	1.44 (0.64–3.53)	0.395	1.13 (0.70–1.83)	0.619
75–84	1.87 (1.24–2.85)	0.003	2.78 (1.27–6.61)	0.014	1.60 (0.97–2.67)	0.070
≥85	2.42 (1.58–3.73)	<0.001	3.61 (1.64–8.63)	0.002	1.90 (1.10–3.32)	0.023
CHA_2_DS_2_‐VASc	1.02 (0.94–1.10)	0.657	1.01 (0.90–1.12)	0.905	1.03 (0.92–1.15)	0.645
Modified HAS‐BLED	1.07 (0.94–1.22)	0.297	1.07 (0.87–1.30)	0.528	1.07 (0.90–1.28)	0.421
Prior OAC purchase						
None	Reference		Reference		Reference	
DOAC	0.92 (0.67–1.26)	0.606	1.10 (0.71–1.71)	0.660	0.75 (0.47–1.20)	0.242
Warfarin	1.05 (0.87–1.28)	0.603	1.08 (0.82–1.41)	0.598	1.01 (0.75–1.35)	0.957
Warfarin vs DOAC	1.15 (0.84–1.57)	0.400	0.97 (0.63–1.50)	0.908	1.34 (0.83–2.17)	0.233
Event year						
2009–2011	Reference		Reference		Reference	
2012–2014	0.82 (0.58–1.16)	0.260	1.07 (0.65–1.78)	0.781	0.65 (0.40–1.04)	0.073
2015–2018	0.68 (0.49–0.94)	0.021	0.84 (0.52–1.37)	0.490	0.57 (0.37–0.89)	0.013

DOAC indicates direct oral anticoagulant; OAC, oral anticoagulant; and OR, odds ratio.

## DISCUSSION

In this study, the main findings were that despite a notable increase in the prior use of OACs, primarily DOACs, the incidence rate of first‐ever HS and 30‐day case fatality remained stable among patients with AF. Importantly, no associations were found between prior OAC use and case fatality in the multivariable models. Older age emerged as a significant factor strongly associated with both incidence and case fatality, with an increasing proportion of HS patients >84 years of age. These findings suggest a favorable trend with respect to the survival of patients with AF suffering from HS, despite the growing number of older patients with AF on OACs.

To our knowledge, this is the first study to report the incidence rate and case fatality of first‐ever HS in an unselected population with AF, including nonhospitalized patients with HS as a cause of death. Comparisons with existing literature should be made cautiously due to variations in study designs, inclusion criteria, and treatment options over time.^20^, ^22^, ^23^, ^24^, ^25^, ^26^, ^27^ In this study, we decided to concentrate on nontraumatic intracranial bleeding and restricted the outcome to nontraumatic ICHs and SAHs as earlier defined to form HSs. In line with the definition, we excluded subdural hemorrhages as although sometimes nontraumatic, they most typically are associated with some trauma, which can be difficult to verify, especially within registry‐based design.^1^, ^38^ Previous population‐based studies on AF have mainly reported overall intracranial bleeding and typically initiated follow‐up from OAC initiation.^28^, ^29^, ^30^, ^31^, ^32^, ^41^ Most of these studies relied on hospital records for case identification, which may have led to an underestimation of the incidence due to the absence of nonhospitalized cases.^20^, ^24^, ^25^, ^26^ Conversely, many studies do not report the exclusion of prior intracranial bleeding events, potentially leading to an overestimation of incident cases. A meta‐analysis covering studies from 1998 to 2010 reported wide variation in intracranial bleeding IRs among patients with AF who were not receiving OAC therapy, ranging from 0.06 to 0.94 per 100 patient‐years, with a pooled IR of 0.32.^23^ Other studies have reported annual rates between 0.2% and 1.0% for OAC‐related intracranial bleeding among patients with AF, with lower rates associated with DOAC use.^22^, ^24^, ^25^, ^27^, ^28^, ^29^, ^31^, ^32^ Similarly, in our previous report, we reported a lower incidence of all intracranial bleeding events in patients on DOACs than in those on warfarin.^41^ In this study, we report an average annual IR of 2.8 per 1000 patient‐years for all first‐ever HS cases in an unselected population with AF, aligning with the literature.

Our study did not find a clear temporal trend in the incidence of HS in the population with AF. Recent studies in the general population have shown decreasing rates in ICH and SAH occurrence,^12^, ^13^ whereas others have reported more stable incidences in recent years.^1^, ^19^ For example, a Finnish study reported a decrease in SAH incidence from 10.2 to 6.9 per 100 000 people between periods 1998 to 2000 and 2015 to 2017.^13^ Notably, our patients with AF and SAH were older, with an average age of 77 years, than the nationwide cohort with SAH, which had an average age of 58 years. This age difference suggests that the contribution of SAH causes may vary between study populations. In addition, we included all I60* codes (Figure S2), potentially capturing more nonaneurysmal SAH cases, with amyloid angiopathy being a typical cause in older people.^42^, ^43^, ^44^ Regarding ICHs, a Danish population level study reported a decreasing incidence of first‐time hospitalizations for spontaneous ICH in the general population 2004 to 2017, particularly among patients >69 years of age.^12^ However, during this period, the proportion of patients with AF increased from 12.4% to 20.7%. Similar results were also reported in a French population‐based stroke registry study, which revealed an increasing proportion of AF among patients with first‐ever ICH from 2006 to 2017, with the prevalence rising from 17.2% to 25.8%.^18^ This increase in AF prevalence among patients with ICH together with a decrease in overall ICH hospitalizations is consistent with our finding of a stable incidence rate of HS in the population with AF.

We observed an increasing proportion of patients with OAC use preceding HS, which agrees with the overall increase in OAC use among patients with AF reported in recent studies.^10^, ^11^, ^18^ A previous study on the general FinACAF population reported a substantial increase in OAC initiation within 1 year following the incident AF diagnosis, rising from 43.6% to 76.3% from 2007 to 2017, with a notable surge driven by the emergence of DOAC use from 2015 onwards.^10^ Similarly, a Danish study reported a rise in OAC initiation from 40% to 50% to 66.5% among patients with incident AF between 2010 and 2015, with a significant preference for DOACs, constituting 72.5% of OAC initiations by 2015.^45^ However, this study described prior event OAC usage only among patients with AF who suffered HS and does not report OAC use in entire population with AF. Earlier studies have reported that older, particularly frail patients are more commonly without an OAC regimen. We presume that the patients who suffered a HS are more likely to belong to this group than the average patient with AF as these factors are also associated with bleeding risk. In addition, it is likely that also other bleeding risk factors are more common and may have influenced the decision to withhold from OAC regimen.^3^, ^4^, ^5^, ^6^

Less is known about HS fatality particularly among the population with AF and including also those without OAC regimen. In the ATRIA (Anticoagulation and Risk Factors in Atrial Fibrillation) cohort from Northern California, 30‐day mortality rates after intracranial hemorrhage among those using OAC were 59% and 42% among those without OAC.^21^ However, the results are from 1996 to 2003. In our study, the average 30‐day CFR after first‐ever HS was 45.4%, without clear temporal trend or significant difference based on prior event OAC use. Neither did we observe significant temporal trends when average annual CFRs were stratified by age groups. Recent studies in the general population have shown decreasing 30‐day mortality of both ICH and SAH. Meta‐analysis from 1980 to 2008 reported a stable median case fatality rate of 40% after ICH^46^ and more recent systematic review of population‐based SAH studies reported decline in the short‐time CFR between 1980 to 2020 an average of −1.5% per year, but with substantial variation between the studies and few high quality studies reporting about temporal trend.^47^ In recent Finnish studies, declining trend in 1‐month mortality was observed for both ICH and SAH. Overall 1‐month mortality for hospitalized patients with ICH was 28% (2004–2018)^16^ and 39% for patients with aneurysmal SAH, including nonhospital deaths (1998–2017).^15^ Among patients with SAH the decline was strongest among the young and middle‐age hospitalized women, but not in the older hospitalized men. In a Danish study on hospitalized patients with ICH, the crude 30‐day mortality declined from 2004 to 2017, but after adjusting for patient characteristics, the trend was no more significant.^12^

Several factors may contribute to this difference in short‐time mortality trend seen in the general population and the population with AF. In line with previous studies,^15^, ^17^, ^21^, ^29^, ^46^ older age was significantly associated with both higher IR and mortality, highlighting the vulnerability of older patients with AF. Furthermore, the average age is consistently lower in general population studies compared with our cohort.^12^, ^15^, ^16^, ^17^, ^18^ A study reporting ICH cases in the Dijon Stroke registry between 2006 to 2017 showed that patients with ICH with AF were older throughout the period, had more comorbidities, and had greater severity at ICH onset compared with other hospitalized patients with ICH. Most OAC users had AF.^18^ Additionally, early withdrawal of active care, a well‐known predictor of increased mortality after HS, may be more common among older patients with AF and could affect early deaths.^17^, ^48^ It is also possible that a greater number of patients with AF and HS die without hospitalization and are therefore not included in studies of hospitalized populations. It is possible that whereas general decreasing trend in short‐term mortality of SAH and ICH has been attributed to improvements in stroke prevention, HS diagnostics, and acute care,^12^, ^16^, ^47^ largely age‐related burden of bleeding risk, increasing OAC usage and older age accumulates in the aging population with AF and could explain the declining trend has not been seen in our population with AF. However, our study did not find association between prior OAC use and 30‐day case fatality rate after first‐ever HS but because this study focused on temporal trends without controlling for confounding factors such as stroke severity and patient characteristics, future research should also examine the various factors influencing clinical decisions and adherence to OAC treatment.

Our study has significant implications for clinicians and policymakers. Although causal interpretations cannot be drawn from this study, our findings suggest that recent advancements in stroke prevention and care—including but not limited to, the increased use of OACs, alongside the decreased use of warfarin—have not led to an increase in HS incidence nor short‐term mortality after HS. However, the increasing absolute number of patients with AF suffering and surviving HS underscores the need for evidence based primary and secondary prevention, rehabilitation, and long‐term care services. In the general population, only 20% to 39% of survivors of ICH regain their previous level of independence,^46^, ^48^ and this proportion may be even lower among patients with AF.

Several important limitations should be acknowledged when interpreting the results of our study. Most important, reliance on registry data exposes the study to potential coding errors and misclassification biases. However, we used at least 5‐year look‐back period for exclusions to distinguish incident cases from prevalent and recurrent cases.^34^, ^35^, ^36^ Additionally, we applied a rigorous algorithm to distinguish actual nontraumatic HS from ischemic events and traumatic intracranial bleedings. Despite these efforts, the use of administrative data has limited the ability to assess certain clinical variables, such as classification of outcome events by cause, stroke severity, treatment decisions, and functional outcomes, which could have provided further insights into HS prognosis. Out‐of‐hospital fatal cases may have been underrepresented, particularly among those with severe comorbidities or high dependency on others. Also, it is possible that AF burden could associate with the HS risk, but unfortunately, we could not reliably distinguish AF types due to our registry‐based study design. Using OAC purchases as surrogate for OAC use is common in registry‐based studies but introduces bias to reported OAC use. To reduce bias, we included only purchases within 90 days before the event, as in Finland, the maximum amount of medication that can be purchased at once is based on 3 months of regular use. In general, caution should be exercised in making causal interpretations or comparisons, as this study was designed to investigate occurrences and trends rather than associations and is inherently descriptive. As our data were collected from 2009 to 2018, it is possible that some temporal trends have developed later. However, we think that our study from that period is useful for clinicians today as it shows that although the changes in stroke prevention presents as higher prior usage of OACs and DOACs among patients with HS, it does not manifest as increased incidence or case fatality among these patients. Despite these limitations, our study has several strengths. The most important factor is the use of a large, population‐based nationwide cohort of patients with incident AF, which enhances the reliability and generalizability of our findings. As HS is typically a severe event that is typically diagnosed in hospitals or through autopsy, our study used both national hospital and death registries to identify cases, including also those without hospitalization. The registries have been previously validated, with a sensitivity of first stroke diagnosis of 95% for patients with SAH and 94% for patients with ICH.^39^

### Conclusions

In conclusion, our population‐based nationwide study found a stable HS incidence rate and short‐term case fatality rates among patients with incident AF over the past decade in Finland. These findings were observed regardless of the significant increase in the prior use of OACs, particularly DOACs, and the increasing proportion of older patients among those with AF who experienced HS.

## Sources of Funding

Paula Tiili; personal research grants from Otto A Malm Foundation, Wilhelm and Else Stockmann Foundation, and Aarne and Aili Turunen Foundation. The FinACAF study has been funded by Helsinki and Uusimaa Hospital District research fund (grant numbers TYH2019309, TYH2023319), The Finnish Foundation for Cardiovascular Research, Aarne Koskelo Foundation, and Sigrid Juselius Foundation.

## Disclosures

Paula Tiili: Research grant: Otto A Malm Foundation, Wilhelm and Else Stockmann Foundation, Aarne and Aili Turunen Foundation. Mika Lehto: Consultant: BMS‐Pfizer Alliance, Bayer, Boehringer‐Ingelheim, and MSD; Speaker: BMS‐Pfizer Alliance, Bayer, Boehringer Ingelheim, MSD, Terve Media and Orion Pharma. Research grants: Aarne Koskelo Foundation, The Finnish Foundation for Cardiovascular Research, and Helsinki and Uusimaa Hospital District research fund. Olli Halminen: none. Jari Haukka: Consultant: Research Janssen R&D; Speaker: Bayer Finland. Ossi Lehtonen: Research grant from Finnish Brain Foundation. Miika Linna: Speaker: BMS‐Pfizer Alliance, Bayer, Boehringer‐Ingelheim. Aapo Aro: Research grant from the Finnish Foundation for Cardiovascular Research. Pirjo Mustonen: Consultant: Roche Diagnostics. Juha Hartikainen: Research grants: The Finnish Foundation for Cardiovascular Research, EU Horizon 2020, EU FP7. Advisory Board Member: BMS‐Pfizer Alliance, Novo Nordisk, Amgen. Speaker: Cardiome, Bayer. K.E. Juhani Airaksinen: Research grants: The Finnish Foundation for Cardiovascular Research; Speaker: Bayer, Pfizer and Boehringer‐Ingelheim. Jukka Putaala: Speaker: Bayer, Boehringer‐Ingelheim, BMS‐Pfizer, Abbott; Advisory board: Novo Nordisk, Herantis Pharma; Visiting editor: Terve Media; Stock ownership: Vital Signum.

## Supporting information

Data S1
Tables S1–S5
Figures S1–S6
