# Supplementary material for: Hemorrhagic Stroke in Atrial Fibrillation: Trends in Incidence, Case Fatality, and Prior Oral Anticoagulation
Source: J Am Heart Assoc. 2025 Jun 11;14(12):e040360. doi: 10.1161/JAHA.124.040360 (PMC12229128; doi:10.1161/JAHA.124.040360)
Supplement: Supplementary file 1 — Data S1 Tables S1–S5 Figures S1–S6 [file JAH3-14-e040360-s001.pdf]

# Supplemental Material

**Data S1. SUMMARY OF THE MAIN ANALYSES CONDUCTED SEPARATELY FOR PATIENTS WHO SUFFERED SAH OR ICH.**

**Patient characteristics and prior first-ever hemorrhagic stroke OAC usage**

Compared with those who suffered ICH, patients with SAH were, on average, younger (77.3 vs 80.7 years), more often female (56% vs 53%) and had marginally lower risk scores. During the observation period, the temporal trends in patient characteristics were similar between patients suffering from ICH and SAH. However, the trend towards increasing proportion of those with higher age was only visible among ICH patients.

Patients with SAH had less often prior OAC purchase than patients with ICH (44% vs 51%). Among those with SAH and ICH, 13% and 11% used DOACs, respectively. The distribution of OAC usage across age groups was similar among both HS types. Additionally, temporal trends in prior OAC use were similar to those in the overall population but less prominent among those with SAH.

**Incidence rates and their temporal trends**

We did not find a significant time trend in IRs of ICH, crude or age-standardized rates. The average annual IR of ICH was 2.44 (95% CI 2.34-2.54) with a range of 2.21 to 2.82. In the Poisson regression model, advancing age, male sex and latest cohort entry year were associated with higher IR. Neither did we find temporal trends in SAH IR, and although increased age was associated with IR, the association was not a s strong a s with ICH. The average annual IR for SAH was 0.38 (0.34-0.42) with the range of 0.27 to 0.49.

Among both types, women had higher crude IRs compared to men (2.60 [2.43–2.78] vs 2.25 [2.09–2.41] and 0.42 [0.35–0.50] vs 0.32 [0.27–0.39]). IRs increased with age. For SAH, IR was highest among women aged 75–84 years, 0.56 (0.43–0.72), whereas for ICH, among women aged over 84 years, it was 4.11 (3.69–4.56). The age-standardized ICH IRs for women and men were 2.25 (2.1–2.41) and 2.59 (2.40–2.79), respectively, and those for SAH patients were 0.40 (0.33–0.47) and 0.35 (0.29–0.43), respectively.

**30-day case fatalities and their temporal trends**

During the observation period, the ICH CFR was highest in 2011 (54.4%) and then decreased to 42.1% in 2017 and 43.7% in 2018. The annual average ICH 30-day CFR was 47.0% (44.7%–49.3%), ranging from 42.0% to 53.8%. The overall 30-day CFR was significantly higher for women than for men (48.6% [45.2%–51.9%] vs 43.2% [39.7%–46.8%], *P*=0.03). Increasing age was significantly associated with increased CFR, ranging from 32% to 54% between the youngest and oldest age groups. According to the multivariate analysis, only increased age was significantly associated with higher CFR.

The 30-day CFR after SAH fluctuated without trend over the years. Average annual CFR was 43.5% (36.7%-50.2%) and ranged from 25.8 to 62.6% reflecting the low number of cases per year. Among patients with SAH, there was a significant difference in the 30-day CFR among women (48%) and men (33%), with an overall CFR of 41.2% (35.2–47.6) (*P*=0.025). Increasing age was significantly associated with higher CFR (*P*=0.003), particularly among women. Among all SAH patients, the CFR was lowest among women aged 20–64 years (n=2/11, 18%) and highest among those over 84 years (n=24/36, 67%). Prior OAC usage was not associated with the CFR.

**Figure S1.** Study cohort selection flow chart.

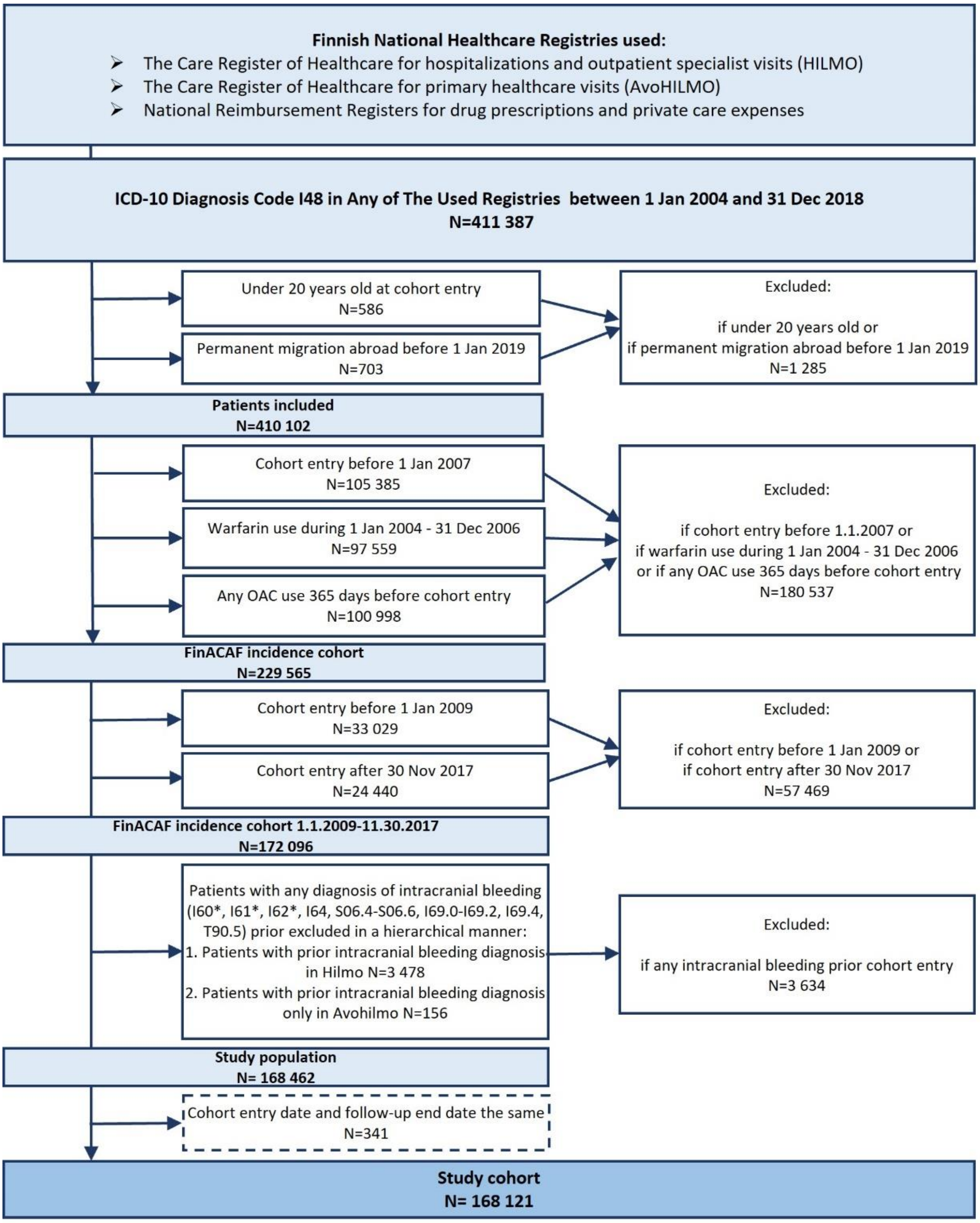

**Figure S2.** Case identification flow chart.

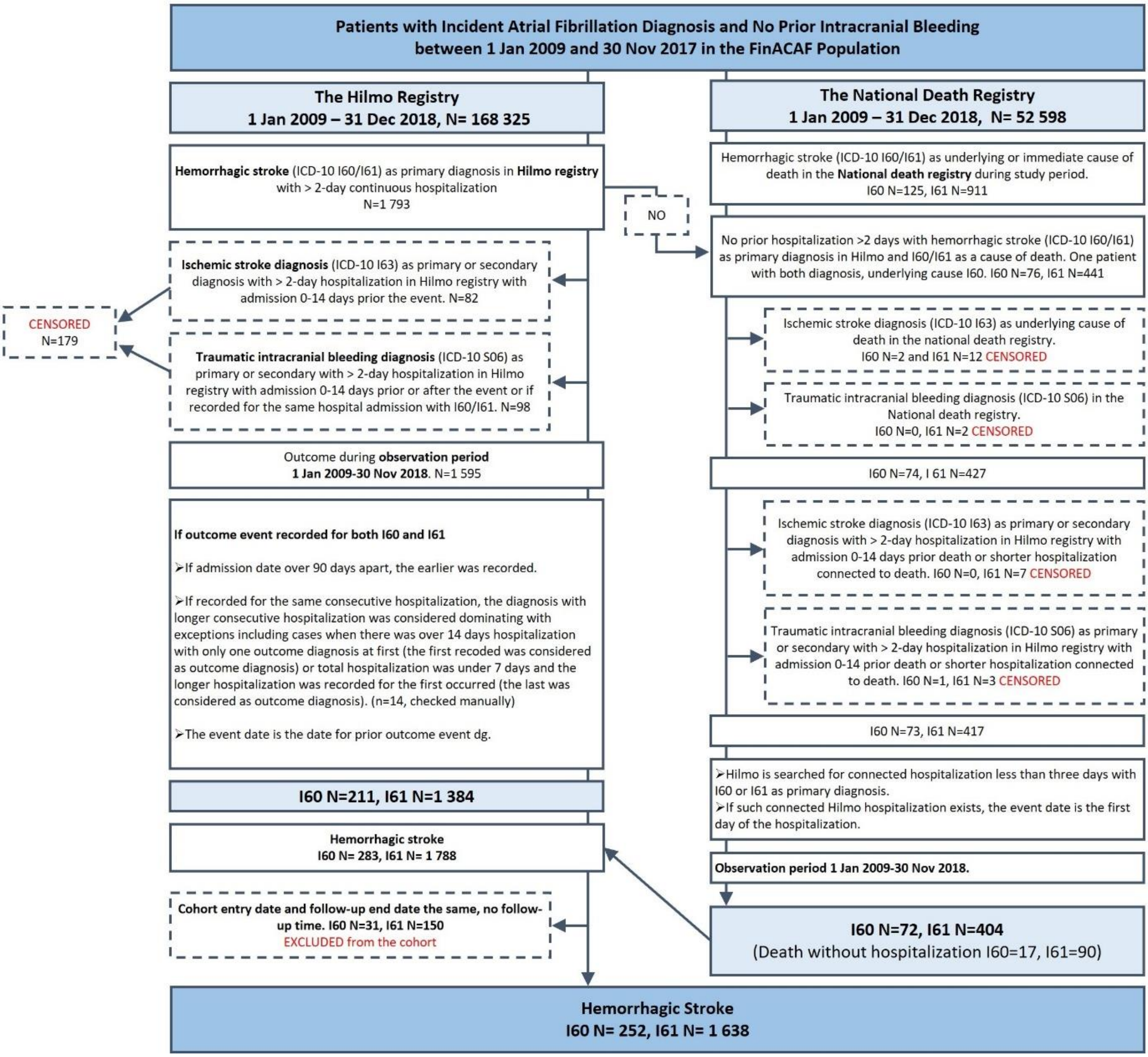

**Figure S3.** Temporal trends in age distribution among patients with AF and first-ever hemorrhagic stroke (All, Women, Men).

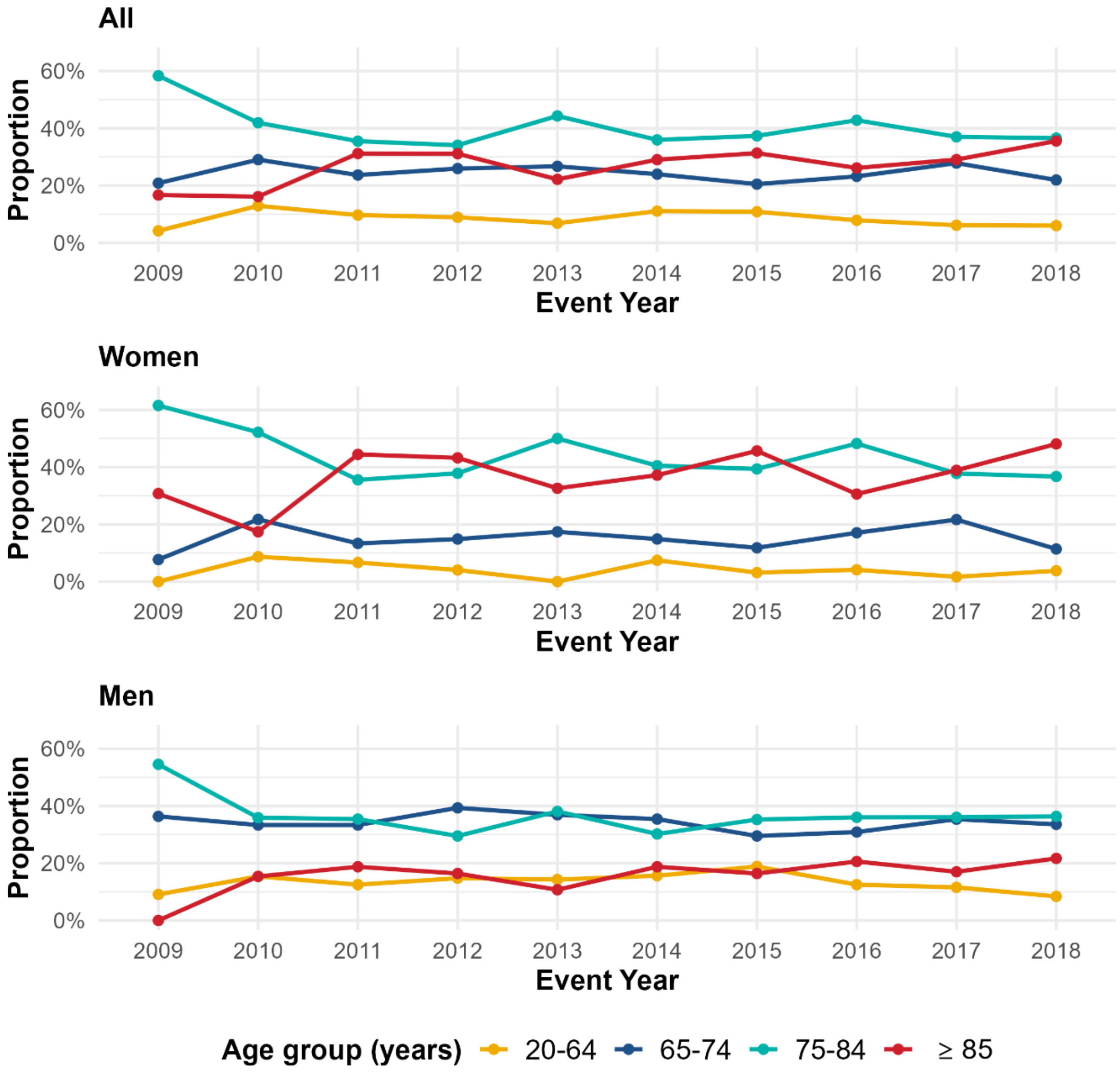

**Figure S4.** Temporal trends in anticoagulant purchases within 90 days prior to first-ever hemorrhagic stroke among patients with atrial fibrillation, stratified by sex.

DOAC = Direct oral anticoagulant, LMWH = Low-molecular-weight heparin.

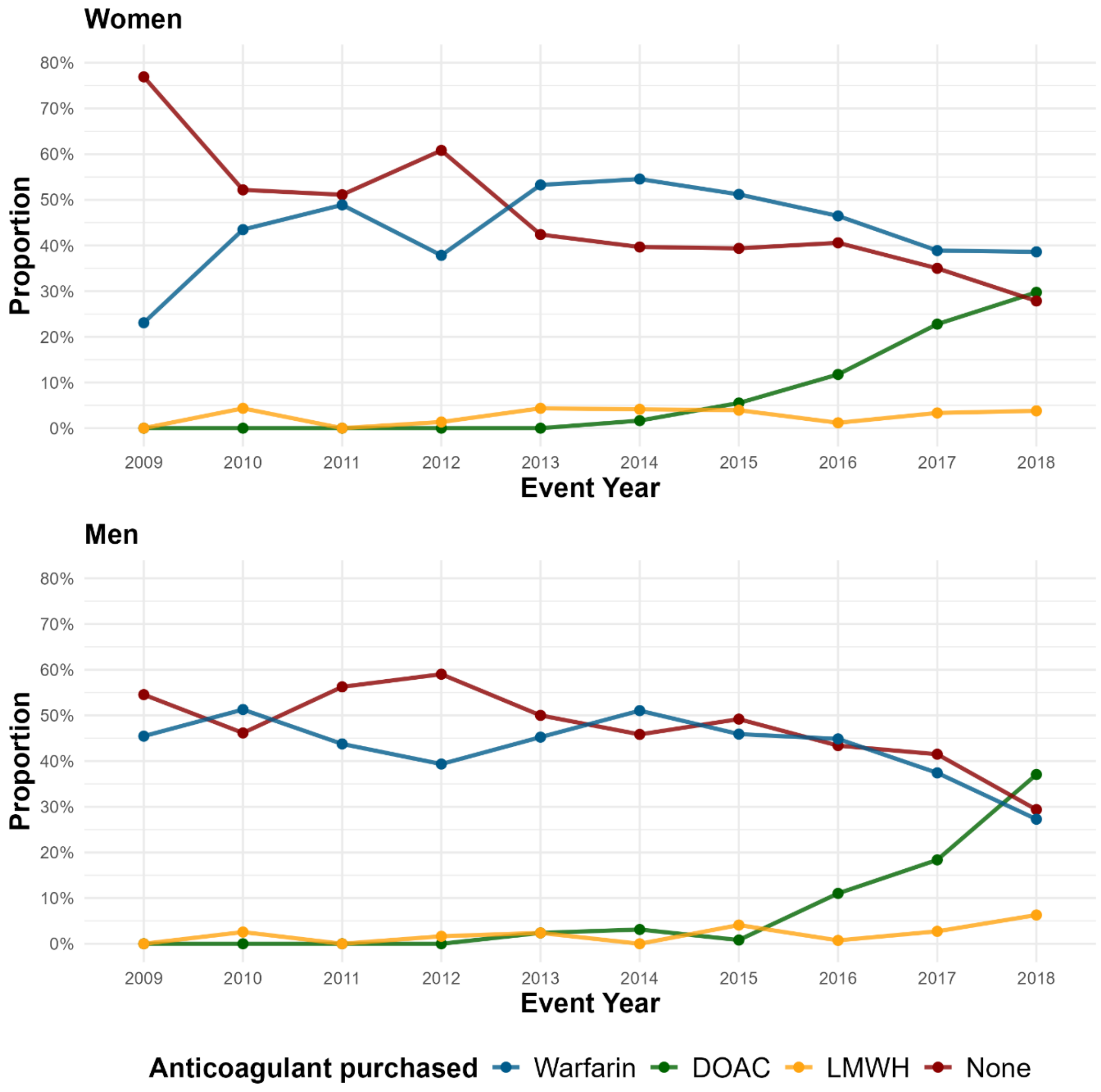

**Figure S5.** Annual crude incidence rates (per 1000 patient years) of first-ever hemorrhagic strokes (bars) among patients with AF and annual proportions of the patients with preceding OAC use (lines).

OAC = oral anticoagulant, DOAC = Direct oral anticoagulant.

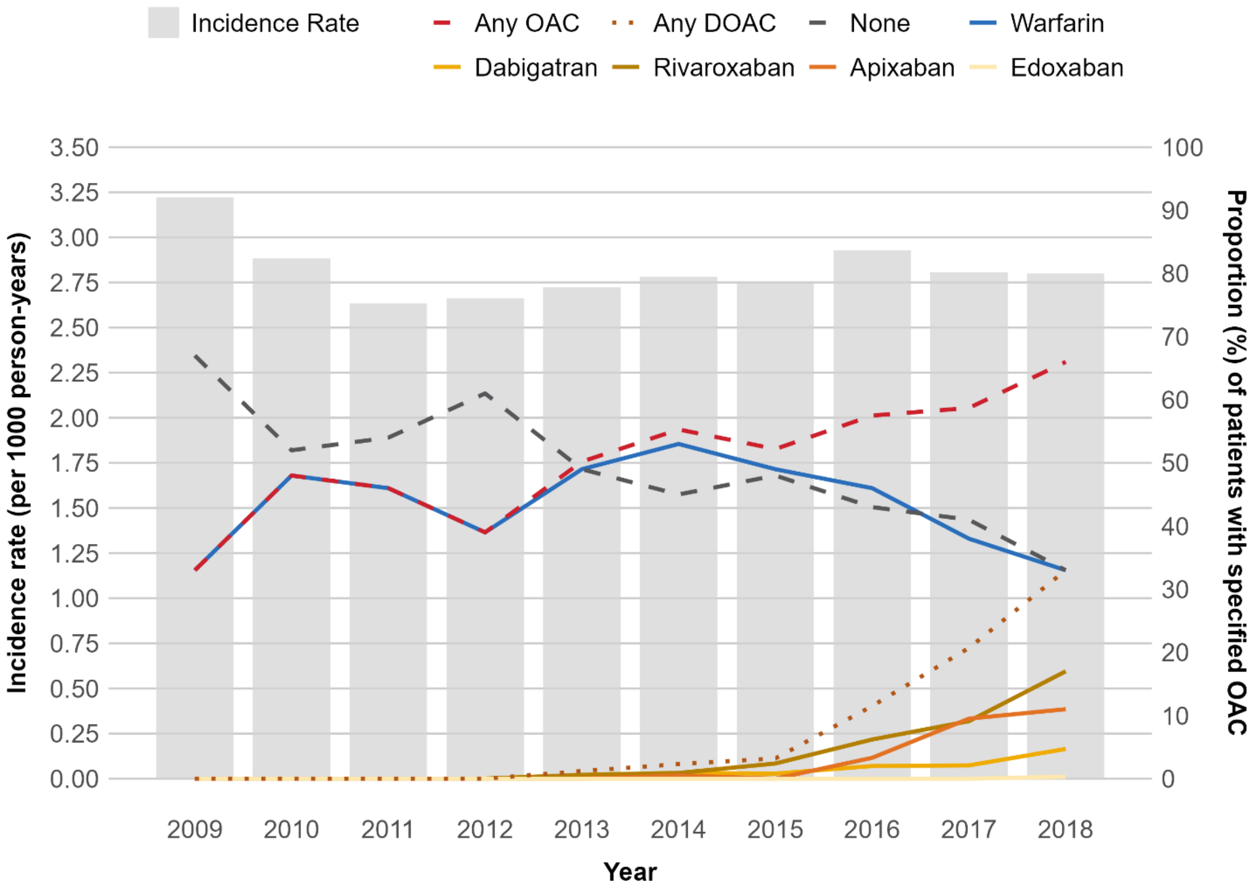

**Figure S6.** Average annual 30-day case fatality rates after hemorrhagic stroke, stratified by age groups. The shaded areas denote 95% confidence intervals.

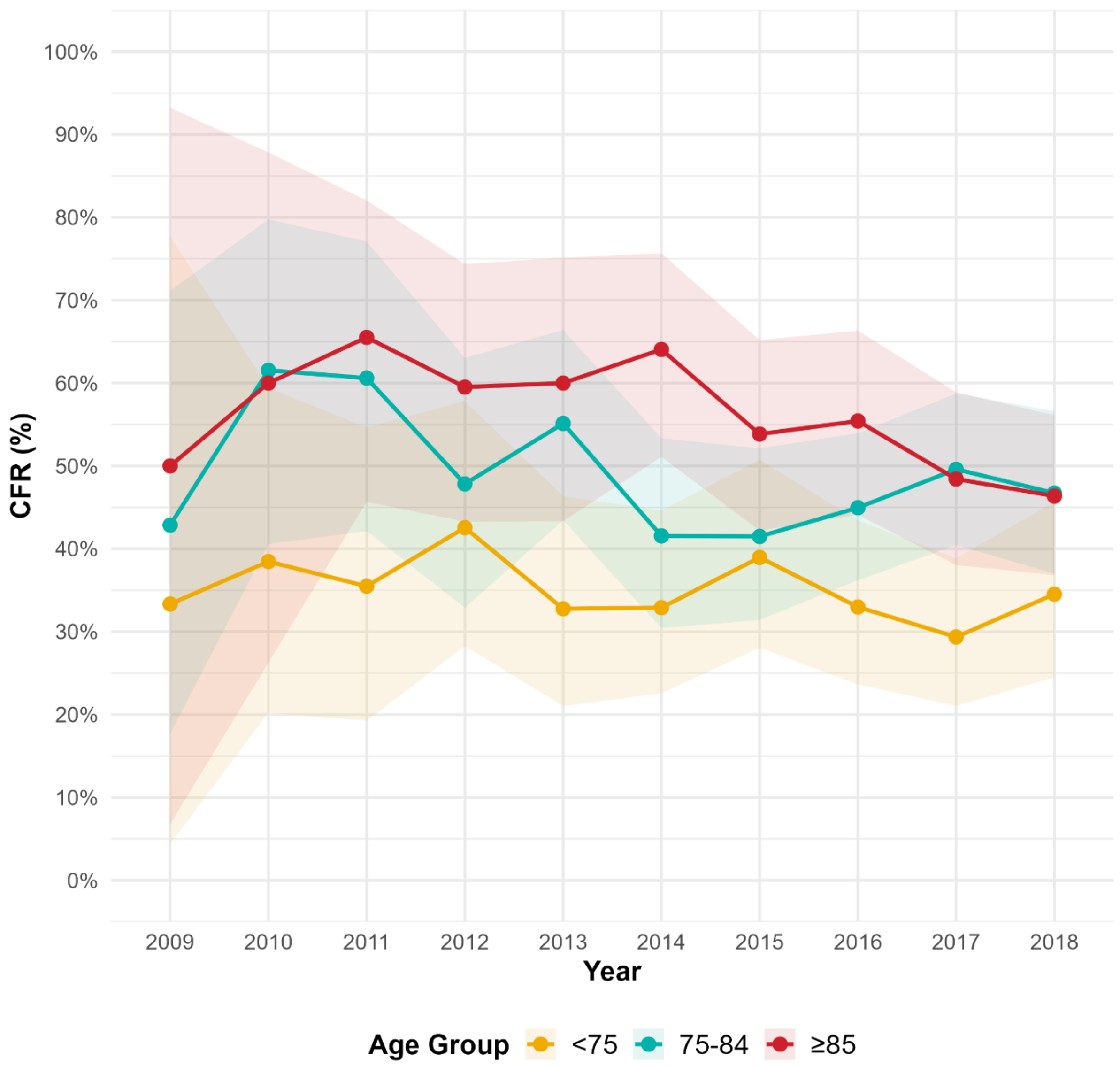

**Table S1.** Diagnosis codes used for morbidity definitions.

| Morbidity | ICD-10 diagnosis codes |
|---|---|
| Atrial fibrillation | I48 |
| Previous intracranial bleeding | I60, I61, I62, I64, S04-S06.6, I69.0-I69.2, I69.4 |
| Intracerebral hemorrhage | I61 |
| Subarachnoid hemorrhage | I60 |
| Traumatic intracranial bleeding | S06 |
| Congestive heart failure | I50, I11.0, I13.0, I13.2 |
| Hypertension | I10-I15 |
| Diabetes | E11-E14 |
| Previous stroke | I63, I64, I69.3-I69.8 |
| TIA | G45 |
| Vascular disease | I20-I25, I65-I66, I67.2, I70 |
| Abnormal renal function | N18, Z49 |
| Abnormal liver function | K70.2-K70.4, K71.7, K71.8, K72, K74 |
| Bleeding history or predisposition | D50.0, D62, D68.3, I62, I85.0, I86.4, J94.2, K22.1, K22.3, K22.6, K25.0, K25.2, K25.4, K25.6, K26.0, K26.2, K26.4, K26.6, K27.0, K27.2, K27.4, K27.6, K28.0, K28.2, K28.4, K28.6, K29.0, K62.5, K63.1, K63.3, K92.0-K92.2, N02, R04, R31, R58, S06.3-S06.6, S06.8 |
| Alcohol abuse | F10 |

ICD-10 = International Classification of Diseases, Tenth Revision

**Table S2.** ATC-codes for medication definitions.

| Medication | ATC codes |
|---|---|
| Warfarin | B01AA03 |
| Dabigatran | B01AE07 |
| Rivaroxaban | B01AF01, B01AX06 |
| Apixaban | B01AF02 |
| Edoxaban | B01AF03 |
| Concomitant antiplatelet drug | B01AC04, B01AC22, B01AC24, B01AC07, B01AC06 |

ATC = anatomic therapeutic chemical

**Table S3.** Cohort baseline characteristics over cohort entry years from 2009 to 2017.

| Characteristic | Overall N = 168 121 | 2009 N= 15 929 | 2010 N = 15 926 | 2011 N = 18 413 | 2012 N = 18 817 | 2013 N = 19 370 | 2014 N = 19 304 | 2015 N = 19 556 | 2016 N = 21 047 | 2017 N = 19 759 | P value |
|---|---|---|---|---|---|---|---|---|---|---|---|
| **Sex** | | | | | | | | | | | 0.016 |
| **Female** | 84 357(50%) | 8 119(51%) | 8 142(51%) | 9 260(50%) | 9 427(50%) | 9 642(50%) | 9 747(50%) | 9 811(50%) | 10 432(50%) | 9 777(49%) | |
| **Male** | 83 764(50%) | 7 810(49%) | 7 784(49%) | 9 153(50%) | 9 390(50%) | 9 728(50%) | 9 557(50%) | 9 745(50%) | 10 615(50%) | 9 982(51%) | |
| **Age** | 74.3 (65.0–82.5) | 73.8 (63.1–82.2) | 73.6 (63.1–82.3) | 74.1 (64.1–82.5) | 74.2 (64.6–82.5) | 74.1 (64.7–82.4) | 74.2 (65.3–82.6) | 74.4 (66.1–82.5) | 74.8 (66.5–82.7) | 74.7 (66.8–82.8) | <0.001 |
| **Age groups** | | | | | | | | | | | |
| **20-64** | 42 094(25%) | 4 646(29%) | 4722(30%) | 4991(27%) | 4905(26%) | 4977(26%) | 4680(24%) | 4403(23%) | 4600(22%) | 4170(21%) | <0.001 |
| **65-74** | 45 732(27%) | 3 796(24%) | 3852(24%) | 4691(25%) | 4982(26%) | 5235(27%) | 5472(28%) | 5725(29%) | 6101(29%) | 5878(30%) | |
| **75-84** | 50 793(30%) | 4 797(30%) | 4680(29%) | 5522(30%) | 5682(30%) | 5822(30%) | 5764(30%) | 5986(31%) | 6512(31%) | 6028(31%) | |
| **≥85** | 29 502(18%) | 2 690(17%) | 2672(17%) | 3209(17%) | 3248(17%) | 3336(17%) | 3388(18%) | 3442(18%) | 3834(18%) | 3683(19%) | |
| **$CHA_2DS_2$-VASc** | 3.0(2.0–5.0) | 3.0(2.0–5.0) | 3.0(2.0–5.0) | 3.0(2.0–5.0) | 3.0(2.0–5.0) | 3.0(2.0–5.0) | 4.0(2.0–5.0) | 4.0(2.0–5.0) | 4.0(2.0–5.0) | 4.0(2.0–5.0) | <0.001 |
| **0** | 10 178(6.1%) | 1 261(7.9%) | 1 173(7.4%) | 1 232(6.7%) | 1 174(6.2%) | 1 172(6.1%) | 1 122(5.8%) | 1 038(5.3%) | 1 085(5.2%) | 921(4.7%) | <0.001 |
| **1** | 18 380(11%) | 2 017(13%) | 2 048(13%) | 2 218(12%) | 2 113(11%) | 2 171(11%) | 2 059(11%) | 1 945(9.9%) | 2 000(9.5%) | 1 809(9.2%) | |
| **≥2** | 139 563(83%) | 12 651(79%) | 12 705(80%) | 14 963(81%) | 15 530(83%) | 16 027(83%) | 16 123(84%) | 16 573(85%) | 17 962(85%) | 17 029(86%) | |
| **Modified HAS-BLED** | 2.0(1.0–3.0) | 2.0(1.0–3.0) | 2.0(1.0–3.0) | 2.0(1.0–3.0) | 2.0(1.0–3.0) | 2.0(1.0–3.0) | 2.0(1.0–3.0) | 2.0(2.0–3.0) | 2.0(2.0–3.0) | 2.0(2.0–3.0) | <0.001 |
| **<3** | 114 935(68%) | 11 390(72%) | 11 352(71%) | 12 970(70%) | 12 891(69%) | 13 287(69%) | 13 135(68%) | 12 992(66%) | 13 916(66%) | 13 002(66%) | <0.001 |
| **≥3** | 53 186(32%) | 4 539(28%) | 4 574(29%) | 5 443(30%) | 5 926(31%) | 6 083(31%) | 6 169(32%) | 6 564(34%) | 7 131(34%) | 6 757(34%) | |

Numbers presented are median (interquartile range) or number of cases (%). CHA2DS2-VASc = congestive heart failure, hypertension, age ≥ 75 years, diabetes, history of stroke or TIA, vascular disease, age 65–74 years, sex category (female); modified HAS-BLED score = hypertension, abnormal renal or liver function, prior stroke, bleeding history, age > 65 years, alcohol abuse, concomitant antiplatelet/NSAIDs (no labile INR, max score 8); OAC = oral anticoagulant

**Table S4.** Hemorrhagic stroke patient characteristics by event year from 2009 to 2018.

| Characteristic | Overall N = 1 890 | 2009 N = 24 | 2010 N = 62 | 2011 N = 93 | 2012 N = 135 | 2013 N = 176 | 2014 N = 217 | 2015 N = 249 | 2016 N = 306 | 2017 N = 327 | 2018 N = 301 | P value |
|---|---|---|---|---|---|---|---|---|---|---|---|---|
| **Sex** | | | | | | | | | | | | 0.370 |
| **Female** | 1 003(53%) | 13(54%) | 23(37%) | 45(48%) | 74(55%) | 92(52%) | 121(56%) | 127(51%) | 170(56%) | 180(55%) | 158(52%) | |
| **Male** | 887(47%) | 11(46%) | 39(63%) | 48(52%) | 61(45%) | 84(48%) | 96(44%) | 122(49%) | 136(44%) | 147(45%) | 143(48%) | |
| **Age** | 80.2 (72.7–86.1) | 80.8 (74.3–83.4) | 76.8 (70.8–82.3) | 80.4 (72.9–86.2) | 79.8 (72.3–86.4) | 79.3 (72.7–84.4) | 80.4 (72.0–86.4) | 80.8 (73.4–87.0) | 80.2 (72.5–85.1) | 79.5 (72.9–86.1) | 81.9 (73.9–87.2) | 0.094 |
| **Age group** | | | | | | | | | | | | 0.089 |
| **20-64** | 155(8.2%) | 1(4.2%) | 8(13%) | 9(9.7%) | 12(8.9%) | 12(6.8%) | 24(11%) | 27(11%) | 24(7.8%) | 20(6.1%) | 18(6.0%) | |
| **65-74** | 458(24%) | 5(21%) | 18(29%) | 22(24%) | 35(26%) | 47(27%) | 52(24%) | 51(20%) | 71(23%) | 91(28%) | 66(22%) | |
| **75-84** | 730(39%) | 14(58%) | 26(42%) | 33(35%) | 46(34%) | 78(44%) | 78(36%) | 93(37%) | 131(43%) | 121(37%) | 110(37%) | |
| **≥85** | 547(29%) | 4(17%) | 10(16%) | 29(31%) | 42(31%) | 39(22%) | 63(29%) | 78(31%) | 80(26%) | 95(29%) | 107(36%) | |
| **$CHA_2DS_2$-VASc** | 4.0(3.0–6.0) | 4.0(3.0–5.0) | 4.0(3.0–5.8) | 4.0(3.0–6.0) | 4.0(3.0–6.0) | 5.0(3.0–6.0) | 4.0(3.0–5.0) | 5.0(3.0–6.0) | 5.0(3.0–6.0) | 4.0(3.0–6.0) | 4.0(4.0–6.0) | 0.594 |
| **0** | 16(0.8%) | 0(0%) | 1(1.6%) | 2(2.2%) | 1(0.7%) | 2(1.1%) | 1(0.5%) | 2(0.8%) | 1(0.3%) | 4(1.2%) | 2(0.7%) | 0.322 |
| **1** | 61(3.2%) | 0(0%) | 2(3.2%) | 5(5.4%) | 9(6.7%) | 6(3.4%) | 5(2.3%) | 13(5.2%) | 7(2.3%) | 8(2.4%) | 6(2.0%) | |
| **≥2** | 1 813(96%) | 24(100%) | 59(95%) | 86(92%) | 125(93%) | 168(95%) | 211(97%) | 234(94%) | 298(97%) | 315(96%) | 293(97%) | |
| **Modified HAS-BLED** | 2.0(2.0–3.0) | 2.0(2.0–3.0) | 2.0(2.0–3.0) | 2.0(2.0–3.0) | 2.0(2.0–3.0) | 2.0(2.0–3.0) | 2.0(2.0–3.0) | 2.0(2.0–3.0) | 2.0(2.0–3.0) | 2.0(2.0–3.0) | 2.0(2.0–3.0) | 0.058 |
| **<3** | 1 103(58%) | 14(58%) | 38(61%) | 57(61%) | 83(61%) | 108(61%) | 142(65%) | 153(61%) | 162(53%) | 173(53%) | 173(57%) | 0.091 |
| **≥3** | 787(42%) | 10(42%) | 24(39%) | 36(39%) | 52(39%) | 68(39%) | 75(35%) | 96(39%) | 144(47%) | 154(47%) | 128(43%) | |
| **Prior OAC purchase** | | | | | | | | | | | | <0.001 |
| **DOAC** | 218(12%) | 0(0%) | 0(0%) | 0(0%) | 0(0%) | 2(1.1%) | 5(2.3%) | 8(3.2%) | 35(11%) | 68(21%) | 100(33%) | |
| **Warfarin** | 821(43%) | 8(33%) | 30(48%) | 43(46%) | 52(39%) | 87(49%) | 115(53%) | 121(49%) | 140(46%) | 125(38%) | 100(33%) | |
| **None** | 851(45%) | 16(67%) | 32(52%) | 50(54%) | 83(61%) | 87(49%) | 97(45%) | 120(48%) | 131(43%) | 134(41%) | 101(34%) | |

Numbers presented are median (interquartile range) or number of cases (%). CHA2DS2-VASc = congestive heart failure, hypertension, age ≥ 75 years, diabetes, history of stroke or TIA, vascular disease, age 65–74 years, sex category (female); modified HAS-BLED score = hypertension, abnormal renal or liver function, prior stroke, bleeding history, age > 65 years, alcohol abuse, concomitant antiplatelet/NSAIDs (no labile INR, max score 8); OAC = oral anticoagulant, DOAC = direct oral anticoagulant

**Table S5.** Crude incidence rates per 1000 person years and adjusted incidence rate ratios between 2009 and 2018 stratified by sex. Separate models to estimate temporal trends by calendar years and by time periods.

| Characteristics | Women | | | | | Men | | | | |
|---|---|---|---|---|---|---|---|---|---|---|
| | Events (N) | PY | IR (95% CI) | IRR (95% CI)* | P value* | Events (N) | PY | IR (95% CI) | IRR (95% CI) * | P value |
| **Crude IR** | 1003 | 331 869 | 3.0 (2.84–3.22) | - | - | 887 | 344 812 | 2.57 (2.41–2.75) | 1.12 (1.02–1.23) | 0.018 |
| **Age group (years)** | | | | | | | | | | |
| **20 – 64** | 37 | 48 844 | 0.76 (0.53–1.04) | Reference | | 118 | 115 933 | 1.02 (0.84–1.22) | Reference | |
| **65 – 74** | 157 | 81 039 | 1.95 (1.66–2.28) | 2.54 (1.80–3.69) | <0.001 | 300 | 107 527 | 2.88 (2.48–3.12) | 2.76 (2.24–3.43) | <0.001 |
| **75 – 84** | 417 | 115 608 | 3.61 (3.27–3.97) | 4.72 (3.42–6.72) | <0.001 | 313 | 87 213 | 3.59 (3.20–4.01) | 3.55 (2.88–4.40) | <0.001 |
| **≥ 85** | 391 | 86 378 | 4.52 (4.09–5.00) | 5.94 (4.30–8.46) | <0.001 | 156 | 34 139 | 4.57 (3.88–5.35) | 4.53 (3.57–5.76) | <0.001 |
| **Cohort entry years** | | | | | | | | | | |
| **2009 – 2011** | 404 | 146 467 | 2.76 (2.50–3.04) | Reference | | 377 | 153 788 | 2.45 (2.21–2.71) | Reference | |
| **2012 – 2014** | 370 | 121 934 | 3.03 (2.73–3.36) | 1.08 (0.93–1.26) | 0.294 | 327 | 125 936 | 2.60 (2.32–2.89) | 1.12 (0.95–1.32) | 0.163 |
| **2015 – 2017** | 229 | 63 469 | 3.61 (3.16–4.11) | 1.32 (1.09–1.59) | 0.004 | 183 | 65 088 | 2.81 (2.42–3.25) | 1.21 (0.99–1.48) | 0.067 |
| **Calendar years** | | | | 0.99 (0.96–1.02)† | 0.640† | | | | 0.95 (0.92–0.98)[2] | 0.003[2] |
| **2009 – 2011** | 81 | 32 580 | 2.49 (1.99–3.11) | Reference | | 99 | 32 077 | 3.09 (2.52–3.77) | Reference | |
| **2012 – 2014** | 288 | 95 618 | 3.01 (2.68–3.39) | 1.15 (0.90–1.49) | 0.280 | 240 | 97 671 | 2.46 (2.16–2.79) | 0.73 (0.57–0.93) | 0.010 |
| **2015 – 2018** | 634 | 203 671 | 3.11 (2.88–3.37) | 1.06 (0.83–1.38) | 0.646 | 548 | 215 063 | 2.55 (2.34–2.77) | 0.67 (0.53–0.86) | 0.001 |

N= number of cases, PY = patient years, IR = incidence rate per 1000 patient years, IRR = incidence rate ratio, CI = confidence interval,

*Model adjusted by sex, age group, and cohort entry and calendar year periods.

†Model adjusted by sex, age group, and cohort entry and calendar years.
